# Urban Influencers: An Analysis of Urban Identity in YouTube Content of Local Social Media Influencers in a Super-Diverse City

**DOI:** 10.3389/fpsyg.2019.02876

**Published:** 2019-12-19

**Authors:** Anne K. van Eldik, Julia Kneer, Roel O. Lutkenhaus, Jeroen Jansz

**Affiliations:** ^1^Erasmus School of History, Culture and Communication, Erasmus University Rotterdam, Rotterdam, Netherlands; ^2^Center for Media & Health, Gouda, Netherlands

**Keywords:** influencers, urban identity, adolescents, super-diversity, micro-celebrities, social media

## Abstract

Influencers belong to the daily media diet of many adolescents. As role models, they have the potential to play a crucial role in the identity construction of their viewers. In the age of social media, such role models may now be found more locally – in the same city – and perhaps with more diverse backgrounds. This may be particularly valuable to adolescents growing up in super-diverse cities, as they are surrounded by a multitude of groups and identities during a life phase in which they have to make sense of who they are and where they belong. Despite the heterogeneity of these identities, there is one thing all have in common: the city they live in. With the city as a common framework, local influencers may be important role models for these adolescents, particularly in negotiating their urban identity. This paper aims toward mapping the ways in which social media can play a role in the negotiation of urban identity among youngsters by investigating how YouTube influencers from a super-diverse city are related to each other online, and how their content relates to the (super-diverse) city of Rotterdam. Findings show that in their videos and on their channel pages, influencers mainly affiliate themselves with the city through having the city as the background and context of the videos, through their involvement with cultural trends (e.g., soccer, hip-hop) that link to the city, and through their affiliation with other local influencers. We argue that influencers may therefore provide their viewers with content that may potentially help their local viewers strengthen their urban identity.

## Introduction

Social media influencers are highly popular among Dutch youth and can safely be assumed to play a crucial role in the everyday life and media diet of adolescents. These influencers are users of social media who have accumulated a large following online, often to such an extent that it has become (part of) their career (Abidin, 2017). Research shows that they are perceived as (micro-)celebrities and play an important role as media idols (Gleason et al., 2017; Martínez and Olsson, 2019). As social media platforms focused on user-generated content (e.g., YouTube and Instagram) have become increasingly popular, so have their content creators. Some of these individuals, in turn, are strongly admired by adolescents, and some have found a tremendous amount of fame with their large amount of followers (Ofcom, 2017). Due to the user-generated content focused character of these platforms, content creation and publication has become available to many regardless of their geographical location or absence of professional materials (Ito et al., 2010; Turner, 2014). As videos can be made and published from the comfort of one’s own bedroom or neighborhood, a variety of influencers have become available from various locations, increasingly reflecting society’s diversity.

The diversity among these influencers may be of importance to adolescents’ development, as influencers function as idols that adolescents can identify with, especially when similar to them (Hoffner, 1996; Gomillion and Giuliano, 2011; Gleason et al., 2017). The local character of the influencer and their videos may also play an important role in this. In *super-diverse cities* (Vertovec, 2007), characterized by great ethnic, cultural and socio-economic diversity, adolescents find themselves surrounded by different groups and identities, trying to make sense of their place among them. Yet, despite all of their differences, one thing all have in common is the city they live in. Previous research on place-identity introduced a city-oriented perspective: urban identity (Proshansky et al., 1983; Lalli, 1992). This entails a sense of belonging to and identification with the city through the everyday experience of this physical and social setting in the life of the individual (Proshansky et al., 1983; Lalli, 1992). This may, for instance, help to differentiate oneself from citizens of other cities (Lalli, 1992). Urban identity can be particularly important to adolescents with a different cultural or national background, for whom it might be easier to identify with the city, than to identify with the country they live in. Our recent survey study among the same target audience has indicated that migrant adolescents indeed experience a higher sense of urban identity compared to non-migrant adolescents (Van Eldik et al., 2019).

Earlier studies have found that social media play a crucial role in identity development (Valkenburg and Peter, 2011; Boyd, 2014; Baym, 2018). The engagement with the content created by influencers and participation in the comment section are ways in which adolescents can negotiate their identity online. Identifying with and feeling good about one’s fellow citizens has previously been found to be positively related with urban identity, and indications have been found that identifying with one’s fellow citizens is, in turn, positively related to personal self-esteem (Lalli, 1992; Van Eldik et al., 2019). However, representations of an urban identity online may also raise questions about who belongs to the city and who gets to represent it. In light of different identities, a polarization of different urban voices might exist, potentially alienating certain groups. It is therefore important to investigate the way in which the city and its citizens are represented, and by whom they are represented.

Moreover, the intensity of following a local influencer has been associated with urban identity and the related social group self-esteem among urban youth, meaning that they felt connected to the city and they felt good about their fellow citizens (Van Eldik et al., 2019). Local influencers may therefore be assumed to play an important role in identifying with the super-diverse city and its citizens. A next step is to seek out who these local influencers popular among adolescents are and what content they produce and distribute. To do so we analyzed local YouTube influencers who were found to be most popular in an earlier survey study among 324 Rotterdam adolescents (Van Eldik et al., 2019). First of all, the online ecology in which the influencers find themselves may play an important role. Channel pages present information about affiliations with other influencers (featured channels and subscriptions), and channel recommendations shown next to the videos are based on similar audiences and themes. Taking into account YouTube’s ecology may thus offer insight into to what extent local influencers present themselves in the context of local networks, as well as the thematic nature of the channels and networks. Therefore our first two research questions are:

RQ1: To what extent are the local influencers of Rotterdam connected to each other through their YouTube affiliations network?

RQ2: How are these local influencers categorized in YouTube’s recommender system, and how are these communities connected to how the city is portrayed?

Secondly, the content of the influencers’ YouTube channels should also be taken into account. In their textual, visual, and audible elements, the videos may present the city or its citizens in a variety of direct or symbolic ways. Influencers may choose to present themselves, their surroundings, and other people in ways that reflect the way they relate to a city. Hence, our third and fourth research questions were:

RQ3: How does the content of these local influencers reflect the super-diverse city of Rotterdam and its citizens?

RQ4: What elements are present in the video that connect these local influencers to the super-diverse city of Rotterdam and its citizens?

To answer these questions, a network analysis and a thematic analysis were conducted. Combined, these analyses aim to demonstrate in what ways the influencers present and represent the city and its citizens, and help understand what adolescents could potentially draw from their online idols in terms of urban identity.

### Theoretical Framework

#### Adolescents, Media, and Identity

Young people are keen users of social media, and the Dutch youth is no different (Kloosterman and Van Beuningen, 2015). In recent years, visually oriented platforms in particular, such as YouTube and Instagram, have gained increasing popularity among young users in the Netherlands, and have surpassed the more ‘traditional’ social networking sites such as Facebook (Kennisnet, 2017; Van der Veer et al., 2017).

Social media have previously been found to play an important role in adolescents’ identity construction (Valkenburg and Peter, 2011; Cover, 2012; Boyd, 2014; Gleason et al., 2017), which is a crucial and central development in this life phase (Erikson, 1968; Delfos, 2013; Valkenburg and Piotrowski, 2017). Social media offer adolescents the opportunity to get in touch with others from a wide variety of backgrounds (Anderson and Jiang, 2018). This diversity of voices, in turn, may help these adolescents construct and negotiate their identities, and serve as a form of validation (Valkenburg and Peter, 2008; Cover, 2012). This may be done by comparing and contrasting themselves with existing identities, as well as through countering existing narratives (Leurs et al., 2018). Identity construction through social media may also hold difficulties, as it is more difficult to separate various audiences (Boyd, 2014), and may cause a decrease in self-concept unity (e.g., Matsuba, 2006; Davis, 2013). With influencers being central players on many social media platforms, their role in the identity construction of adolescents needs further investigation. It is therefore important to know who these influencers are, and what content they produce that adolescents can borrow from.

#### Influencers as Micro-Celebrities

The participatory character of today’s social media affords users to both view existing material of others and upload their own content – what previously were consumers, are now the producers of content as well (Ritzer and Jurgenson, 2010). Some of these users who produce and share user-generated content have gathered a remarkably large following, essentially turning into celebrities. This can be understood as what Senft (2008) has coined *micro-celebrity*, which entails “a new style of online performance that involves people ‘amping up’ their popularity over the web using technologies like video, blogs, and social networking sites” (p. 25). When the activities of these micro-celebrities exceed that of (sometimes partially paid) hobbies, they become social media influencers, which can be understood as its own career trajectory embedded in the professionalized social media ecology (Abidin, 2017). By creating a personal brand on (and sometimes off of) social media and generating a large following and amount of fame, an influencer becomes attractive to businesses for spreading their advertisements among specific target audiences (Hearn and Schoenhoff, 2016). The success of an influencer is strongly connected to their image or self-branding and their connection to the audience. Authenticity, as a key element, is achieved through blurring the boundaries between the influencers public and private life, and maintaining the status of a perceived amateur (Jerslev, 2016; Abidin, 2017; Ferchaud et al., 2018). Next to that, parasociality and engagement with the audience plays a key role in micro-celebrity, where the influencer creates such a relationship with their audience to maintain engagement by means of intimacy, accessibility, and community (Marwick, 2013; Jerslev, 2016; Ferchaud et al., 2018; Usher, 2018). Collaboration with other influencers is also utilized as a form of cross-promotion, that may further help increase their visibility and further their careers, either by means of existing friendships between influencers or the intervention of a multichannel network (MCN) (Grünewald et al., 2014; Lobato, 2016; Ashton and Patel, 2017). Having cross-promotion among influencers appear in a natural and authentic looking fashion may be important to the credibility of the influencer. Such collaborations and affiliations may, in the case of YouTube, not only be visible in videos, but also on channel pages through influencers’ featured channels or subscriptions. Collaboration may thus help viewers to associate one influencer with the other. All in all, networks among influencers are, in different ways, of great importance to the success of their channels.

#### Influencers and Identity Construction

As media idols, influencers can function as role models for adolescents, given that the consumption of media content by influencers may provide not only entertainment, but also examples of identities (Gomillion and Giuliano, 2011; Turner, 2014; Gleason et al., 2017; Lovelock, 2017). Adolescents may engage with the influencer content by means of consuming the content, participating in comment sections, liking or sharing content, and even producing their own content, for instance in response to or based on the content by these influencers (Shao, 2009; Jansz et al., 2015). As in dialog, influencers often address their audience in a direct way in their videos and engage with them in the comment sections, creating the parasocial interaction [i.e., the one-sided feeling among the audience that they have an intimate personal relationship with the media figure or celebrity (Horton and Wohl, 1956)] that is so crucial to their success (Frobenius, 2014; Burgess and Green, 2018). Parasocial relationships, in turn, have been linked to processes of adolescent identity formation (Gleason et al., 2017).

Early adolescents prefer media idols they can identify with – someone with whom they find they have similarities (Hoffner, 1996; Cohen, 1999; Delfos, 2013; Valkenburg and Piotrowski, 2017). Additionally, previous studies have shown that one’s similarity to a role model, for example with respect to gender and ethnicity, is an important predictor of the influence that the role model has on the individual (e.g., Karunanayake and Nauta, 2004; Lockwood, 2006). With the increasing diversity of role models available due to the affordances of social media to easily upload content, adolescents may find influencers that have these similarities. They can find role models with, for instance, a same cultural or socio-economic background, or someone from the same city or neighborhood. Consequently, the content made by these role models may have an important influence on these adolescents’ identity construction.

However, not everyone has access to the materials, technologies and support that make professional production possible (Usher, 2018). With the appearance of amateurism often pursued to maintain an image of authenticity in spite of professionalization (Abidin, 2017), micro-celebrity, or being an influencer, is often misunderstood and oversimplified as something that is available and practiced by many ordinary people: the availability and accessibility of social media to many may give the impression of a ‘bottom up’ character to micro-celebrity (Usher, 2018). Research showed it is a small group of people with a privileged background in particular that tend to dominate online content creation (Brake, 2014). Becoming an influencer might therefore not be possible for everyone, resulting in a select group of individuals that are more likely to dominate most of the content on social media on an influencer level. With this selectivity in an environment where authenticity is key, it is important to question the extent to which the influencers and their content reflect the diversity that can be found in today’s society, particularly in highly diverse environments. This question is particularly interesting in light of the very thing that adolescents and their favorite local influencers may have in common, which is *place*.

#### Urban Identity

Place plays an important role in the establishment of sense of belonging and the construction of identity (Tuan, 1977; Proshansky et al., 1983; Lalli, 1992). Place-identity has been researched from a variety of theoretical traditions and can be understood on various levels of scale (Tuan, 1977; Proshansky et al., 1983; Lalli, 1992). A form of place identity that is specific to the environment of a city, is urban identity (Proshansky et al., 1983; Lalli, 1992). Urban identity is part of one’s self-identity, and results from the everyday experiences of an individual in the context of the city (Proshansky et al., 1983; Lalli, 1992). Providing the backdrop of everyday experiences, the city becomes a symbol for these experiences, as well as an independent entity that provides “a sense of stability and continuity” (p. 294) to the life of the individual (Lalli, 1992). The city, in this sense, “provides an identity-enhancing context for one’s biography, and thus a continuity which is relatively independent from definite (e.g., social) changes” (Lalli, 1992, p. 294). In essence, urban identity deals with the role that the city and its citizens, in physical as well as symbolic form, play in the everyday life of the individual and how this is part of their understanding of who they are.

Urban identity manifests itself in a variety of ways. Identifying with the city and/or its citizens consciously is one of the most direct ways in which urban identity can be recognized (Proshansky et al., 1983; Lalli, 1992; Belanche et al., 2017). Belanche et al. (2017) emphasize the cognitive character of this dimension of urban identity, stressing the awareness of one’s membership to a social group and identifying with it. This social group may of course also include one’s personal social network. Urban identity is also achieved by differentiating the city and/or its citizens from other cities or citizens (Proshansky et al., 1983; Lalli, 1992; Belanche et al., 2017). As the differentiation between the self and others is central to the creation and negotiation of one’s identity in general, citizens can make sense of and negotiate their urban identity by means of comparing to other cities, or perhaps non-urban environments, as well as the characteristics and behaviors typically associated with it (Lalli, 1992). This comparing and contrasting may also contribute to an evaluative level of urban identity, where positive and negative connotations may play an important role (Belanche et al., 2017). This can, in turn, play a role in self-esteem and self-efficacy (Belanche et al., 2017; Van Eldik et al., 2019). Urban identity may also be viewed from a more environmental and symbolic perspective (Proshansky et al., 1983; Lalli, 1992; Belanche et al., 2017). The spatial characteristics signify the everyday living experience of those living in the city (Proshansky et al., 1983; Lalli, 1992). Certain landmarks or other symbols may also be recognized as typical to that city (Belanche et al., 2017). In line with this argument, famous individuals commonly associated with the city may have a similar symbolic function.

To investigate urban identity in the context of Rotterdam, it is crucial to take into account the specific characteristics of the city. Rotterdam is the second largest city of the Netherlands, being only smaller than the capital Amsterdam, with 644.527 inhabitants (Statistics Netherlands, 2019) and it is a super-diverse city (Scholten et al., 2019), which means it is characterized by great diversity on various complexly intertwined levels (Vertovec, 2007). Rotterdam holds a complexity of diversities, not only related to ethnicity, culture, and socio-economic levels, but also migration history, social and living situations, and more. Among the four largest cities in the Netherlands with more than 300.000 citizens, Rotterdam has the highest number of youth with a non-western migrant background (Statistics Netherlands, 2017). It is a majority-minority city, meaning that the majority of citizens has a migration background (Jennissen et al., 2018). It holds more than 200 different nationalities and a large variety of ethnic backgrounds (Jennissen et al., 2018), with 80,742 (12.6%) western migrants and 224,109 (38.2%) non-western migrants in 2018 (Statistics Netherlands, 2018). Socio-economic diversity is also strongly present in Rotterdam, with 20 to 22% of minors living in poverty in 2016 (Hoff, 2017). Moreover, neighborhoods and schools show segregation in terms of socio-economic status and migration background (Herweijer, 2008; Gemeente Rotterdam, 2019; OBI, 2019). This multi-faceted diversity of the city may therefore be crucial in the everyday experience of the city, and the cultural products and understandings that rise from it (Bennett, 1999; Alim and Pennycook, 2007; Georgiou, 2010).

#### Urban Identity and Social Media

Previous research has investigated different ways in which place can play a role in the use of social media and relating identity construction, for instance through investigation of sharing locations on Four Square (Saker, 2017), and other location-based technologies (Schwartz and Halegoua, 2015). As Schwartz and Halegoua (2015) argue, the mentioning of location on social media can be seen as “parts of larger narratives and performances of embodiment and experience of place” (p. 1656). Previous research has also found indications that a high intensity of following influencers from one’s city is associated with a higher level or urban identity (Van Eldik et al., 2019). Adolescents who followed local influencers on multiple channels had a higher urban identity than those who did not. Influencers therefore can be said to play an important role in the construction of urban identity. Influencers may differentiate themselves from people from other cities, emphasize the positive characteristics of the city, or adolescents might recognize themselves in small details with regards to one’s language or habits. The narrative of a local influencer with regards to their background and identity in the context of the city may also invite adolescent audiences to recognize their similarity with (the lives of) the influencer. For instance, a local influencer might move through the city center while recording their vlog, providing the viewer with an environment that is highly recognizable, or might even openly announce that they identify as a “Rotterdammer”. Moreover, the diversity of citizens in the super-diversity may play a crucial and interesting role. Previous research has argued that media idols and influencers can play an important role for minorities (Gomillion and Giuliano, 2011; Briggs et al., 2012; Lovelock, 2017). With fewer role models in traditional media, social media may provide a place where adolescents may find positive role models. Role models have previously been found to stimulate a positive self-image in the context of sex-roles (Ochman, 1996) or may serve as sources of pride, inspiration and comfort for sexual minorities (Gomillion and Giuliano, 2011), yet media may also hold stereotypes that may negatively affect self-esteem, as was found in the context of African–American youths (Ward, 2004). It is therefore interesting to investigate how diverse these role models, who have the city and therefore presumably also urban identity in common, and their representations of the city are. With their expressions of urban identity, influencers may also stimulate that identity in the adolescent audience member. Connection through place may not only be prevalent from the content of their videos, but also from their collaborations and affiliations with, or thematic connection to, other influencers from that same city visible on their channels. As urban identity may be tied to and interconnected with many elements of an individual’s life and experiences, content produced by influencers with explicit or implicit references to the city and its (youth) culture, as well as the connections between these local influencers, may therefore play a crucial role in the negotiation of their viewers’ urban identity.

## Materials and Methods

Two methods were used in this study. First, a small-scale network analysis was conducted based on a previous survey that asked for local influencers (Van Eldik et al., 2019), to help us understand patterns of interest and collaboration between and around these influencers on YouTube (i.e., affiliation network), and to understand their position in thematic communities resulting from YouTube’s recommender systems (i.e., related channel network). Second, a thematic content analysis was conducted on the content of six influencers that were selected on the basis of the survey, focusing on the ways in which the city is present and urban identity is communicated to the audience, both implicitly and explicitly.

### Sample

A survey among 324 Rotterdam adolescents aged 9 to 13 (*n* = 321; *M* = 10.65; *SD* = 0.88), including 160 boys and 159 girls, was conducted in 2018 (Van Eldik et al., 2019). The survey was conducted on three different schools in two different neighborhoods of Rotterdam, representing a multitude of socio-economic, migration, and cultural backgrounds. The survey asked participants for their two favorite influencers from Rotterdam and on which social media platforms they followed them. Non-local influencers or local non-influencers were omitted from the resulting list, and the frequency of mentions of each local influencer was noted. A local influencer was defined as an individual with a social media account from the region of Rotterdam who had a significant following (>15.000 subscribers). This resulted in a list of local influencers with YouTube and Instagram as the main platforms. From here we focused on YouTube, not only for its popularity, but also because, unlike Instagram, the platform allows for retrieval of their channels’ networked context. Currently YouTube is visited by around 2 billion logged-in users each month (Solsman, 2019; YouTube, n.d.). YouTube provides a space where all users can upload videos, which can be viewed by others, with room for ‘liking,’ commenting and sharing the content. The resulting list of 18 influencers, with subscriber counts ranging between 15.000 and 1.500.000 (*M* = 339,016; *SD* = 379,325), was the foundation for the following analyses. This research was carried out with the approval of ESHCC’s Ethics Review Board.

### Network Analysis – Influencer Network

A network analysis was conducted to identify patterns of interest and (local) collaboration (RQ1), and identify larger thematic communities in which the influencers’ YouTube channels are algorithmically categorized (RQ2). First, in order to uncover patterns of interest and collaboration, we used Rieder’s (2015) YouTube Data Tools to retrieve the featured channels and channel subscriptions of the 18 influencers identified by the respondents of the survey, including the 6 influencers that were selected for the thematic analysis. Featured channels are other YouTube channels that users display on their channels with the goal of showing other users’ channels that they recommend, typically signifying affiliation or collaboration. Subscriptions can be found on the same channel profile page, but refer to other YouTube channels that the user follows, highlighting interest and/or identification. Besides the first degree connections, we also retrieved second and third degree connections of the 18 influencers, each time connecting the channels returned by previous iterations with new sets of channels. *Gephi*, a network analysis and visualization software package, was used to visualize the ‘affiliation network’ (Bastian et al., 2009).

Second, in order to understand the influencers’ channel’s position in YouTube’s recommender systems – YouTube typically clusters channels that cover similar themes together (Rieder et al., 2018) – we used Rieder’s (2015) YouTube Data Tools to retrieve the related channels for our 18 influencers. Here, too, we retrieved second and third degree connections. This resulted in our ‘related channels network’ that we also visualized using *Gephi*. We used *Gephi*’s implementation of the Louvain algorithm with a resolution of 0.8 to detect communities in the ‘related channel network’ (Blondel et al., 2008). In contrast to the ‘affiliation network’ that showed patterns of interest and affiliation between channels, this network reflects types of channels associated with each other based on YouTube’s algorithm, likely based on previous user engagement, indicating a similar target audience, and the communities in this network often signify thematic clusters. Further details on both steps can be found in Section “Results.”

### Qualitative Content Analysis – Video Content

In our qualitative content analysis we investigated the representation of the city and the citizens (RQ3), and how the influencer positioned themselves in it (RQ4). We focused on local influencers who made video material in the form of vlogs. Here, we defined vlogs as all user-generated videos that include the maker of the video talking to the camera in a casual fashion. This may entail the influencer taking the viewer with them in their everyday lives, but may also include more structured formats, such as interviewing or challenges.

Six of the local influencers from the survey were selected for further in-depth investigation. In this sample, five out of six influencers were men, and two out of six influencers could be categorized as white Dutch with no apparent migration background, whereas the other four seemed to be of various other ethnic and cultural backgrounds. During the selection of these six influencers, the most frequently mentioned influencers were chosen, influencers who did not produce vlogs were omitted, and in case a local influencer had more than one YouTube channel, only one of these channels was chosen (often their main or individual channel). We selected the 40 most recent vlogs from each influencer for a pre-selection. This resulted in 240 videos that went through a pre-selection process, investigating the extent to which these videos presented the city, its citizens, or urban identity. Moreover, videos that were not considered vlogs were omitted. Videos that were made in high affiliation with a third party (except for other influencers) were also omitted. In total, 134 (56%) videos were deemed relevant enough, and the 10 most recent relevant videos of each channel were selected for further in-depth analysis, leaving a total of 60 videos. We aimed to answer to what extent and how the content published by local YouTube influencers, popular among local adolescents, relates to the super-diverse city (RQ3 and RQ4). Following the thematic analysis approach by Braun and Clarke (2006), we first familiarized ourselves with the data, after which the initial open codes were established. During the next step of looking for themes, 7 themes with 1–9 subthemes each emerged, which were reviewed on the level of the codes and the dataset. This was further limited and merged to a total of 5 themes, with 2–6 subthemes each, which, in turn, were further defined and specified. Two coders were involved in the measurement of the intercoder agreement and the establishment of the codebook (see Supplementary Material for the codebook). The intercoder agreement based on a subsample of the videos had a Krippendorff’s α = 0.81 (Hayes and Krippendorff, 2007).

## Results

### Network Analysis of YouTube Channels

In our network analyses we aimed to position the influencers in relation to each other in an affiliation network, as well as in relation to thematic communities in a related channel network.

#### How Are the Influencers Connected to Each Other?

**FIGURE 1:**
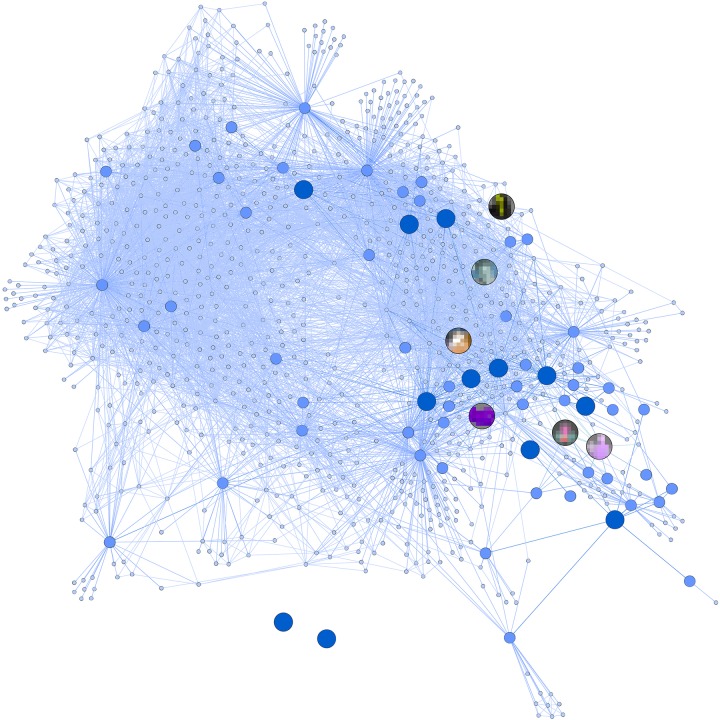
The figure shows the affiliations between channels based on their featured channels and subscriptions. The dark blue dots indicate the original 18 influencers, the medium blue dots indicate the channels directly connected to one of the 18 influencers (1^st^ degree connections), and the light blue dots indicate second degree connections. The six influencers that are analyzed in the thematic analysis are displayed with blurred pictures. Lines between the dots demonstrate a connection between two influencers through their featured channels or subscriptions.

To analyze the patterns of affiliation and interest between the influencers, three steps were taken. First, we analyzed the direct links between the featured channels and subscriptions of the 18 influencers identified in the survey – to what extent they referred to each other on their channel pages. By analyzing the affiliation network, we found that 9 of the 18 influencers were directly linked to each other’s YouTube channels (network density = 0.039), typically demonstrating affiliation and/or interest. This network consists of two components, meaning that there are two clusters of influencers linking to each other with no connection between these groups. The other nine were not connected to any of the original channels at this point. Second, we investigated the featured channels and subscriptions of the channels that the original 18 influencers linked to, to see if there were possible affiliations between the 18 influencers through other channels. We found that these 69 channels had 128 connections between them (network density = 0.027), which were separated into four components, each holding 2 to 50 channels. Three of our original influencers still remained without any links, or affiliations, with other channels. As a final step, we investigated the featured channels and subscriptions that the previous 69 channels’ linked to. We found 937 channels with 7558 connections between them (network density = 0.009). The previously separate components now connected to each other, becoming one component, and only two of the original 18 channels remained without affiliations. Almost all 18 influencers are thus indirectly affiliated with each other within three degrees of separation, contributing to the appearance of a local group of influencers that are familiar with each other (see Figure 1 for a visualization of the previous three steps and their connections). Such a locally connected group may, in turn, strengthen a sense of the city as a shared identity.

#### In What Communities Do the Influencers Position Themselves?

To investigate the position of the 18 influencers in YouTube’s recommender systems, we analyzed the related channel network. The related channels algorithm connects YouTube channels that are engaged with by the same users. On a network level, this leads to communities of channels that are likely to have something in common, such as a similar topical focus. The related channels were gathered in April, 2019. Unfortunately, the related channel feature is no longer available on YouTube since May 2019 (YouTube, 2019). Retrieving first, second, and third degree connections caused the network to grow exponentially with each iteration. This resulted in a network of 905 channels and 4809 connections between them. Community detection resulted in 15 communities, with a modularity score of 0.72. We have reflected on the contents of the channels in each community to label them accordingly. Table 1 gives an oversight of the communities in which our influencers found themselves.

**TABLE 1:** An oversight of the communities in which the original 18 influencers were present.

**Number of original 18 (and 6) influencers**	**Description**
8 (2)	*Dutch Urban Music Community* Mostly Dutch male YouTubers, as well as music channels, with a strong tendency toward urban music (hip hop, R&B, and rap) and with great ethnic and cultural diversity. Targeted toward those interested in urban music.
3 (0)	*Dutch Family (Friendly) Vloggers Community* Mostly Dutch family-friendly lifestyle vloggers, such as teens and families with children. Contains content about everyday life, toys, but also beauty and fashion. Likely oriented at a predominantly younger (female) audience.
2 (1)	*Dutch Soccer Community* Predominantly Dutch soccer vloggers, teams, channels and sponsors. Aimed at soccer enthusiasts.
2 (1)	*Dutch Entertainment and Games Vlogger Community* Mostly Dutch white male vloggers who focus on vlogs, challenges, pranks, humor, extreme sports and games. Likely oriented at early adolescent and teenage boys.
2 (2)	*Dutch Lifestyle Vlogger Community* Dutch YouTubers, vloggers, predominantly white young adult females, addressing lifestyle, beauty and fashion, among other things. Likely oriented at, but not limited to, teenage, or young adult females.

*The six influencers selected for thematic analysis are indicated in between brackets.*

**FIGURE 2:**
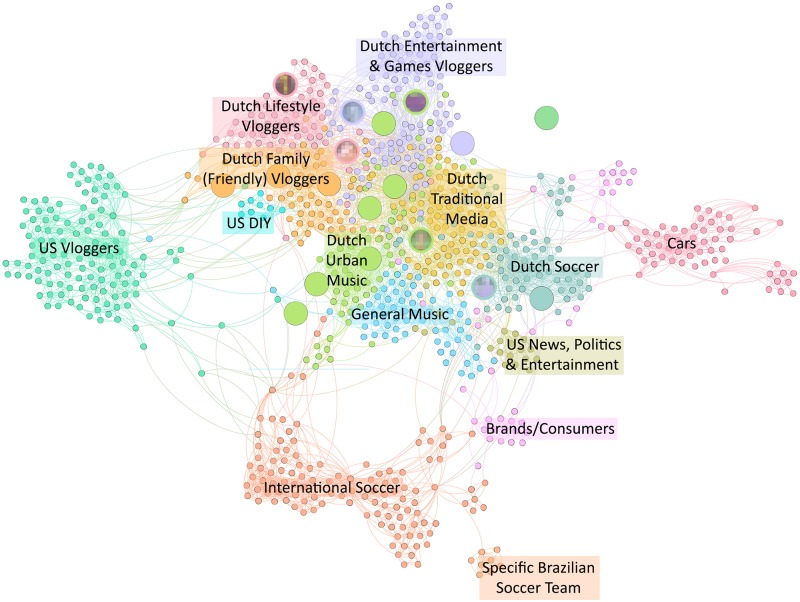
The related channel network including the 15 communities found through community detection. The original 18 influencers are enlarged, and the six influencers part of our thematic analysis contain blurred pictures. The direction of connection is indicated by a clockwise bended line between the dots.

One of the 18 influencers was not attached to any part of the component and had no connections of its own. While we found no clear indications why, it might be caused by the scale of our data collection. More iterations would perhaps connect the channel to the component. Other communities found, but not containing any of our influencers were: *Dutch Traditional Media Community*, *General Music Community*, *US DIY Community*, *US Vlogger Community*, *Car Community*, *Specific Brazilian Soccer Team Community*, *International Soccer Community*, *General Brand/Consumer Community*, and *US News, Politics & Entertainment Community* (see Figure 2 for a visual representation of the different communities, their links, and the positions of our original 18 and 6 influencers in it). Most of these communities were international or predominantly US-based, whereas the communities that included the influencers were all predominantly Dutch.

The communities in which the influencers were found are therefore diverse, but reflect well the interests of the adolescents. Interestingly, almost half of the influencers were located in the *Dutch Urban Music Community*. This is particularly relevant due to the urban and diverse character of this music scene, which fits the super-diverse character of the city. With this and the affiliations between local influencers in mind, it invites further investigation of the content of the videos to establish the ways in which the city, its citizens, and urban identity are present and how they are portrayed.

### Content Analysis of YouTube Videos

In the following section we present our findings of the thematic analysis. Five themes were found, most of them divided into a number of subthemes. The main themes are: *Being and Living in the City*, *Culture and Diversity*, *People and Relationships*, *Urban Symbols*, and *Other Identities.*

#### Being and Living in the City

As a first theme, being and living in the city emerged. We found that there were commonly direct references to living in, being in, or being from Rotterdam. This was generally focused on mentioning one’s whereabouts, showing the environment, and stating one’s roots. This theme can be separated into three subthemes: Being in Rotterdam, Living in Rotterdam, and Being from Rotterdam.

##### Being in Rotterdam

As influencers were followed through their everyday lives, they often pointed out that they were in Rotterdam at various moments of their day being occupied in various activities. This was mostly done by means of saying this to the audience: “I am here, somewhere in a neighborhood in Rotterdam” or “I get my nails done here in Rotterdam.” Another influencer indicated his location in Rotterdam on a map that was later edited into the video. Such statements focus the attention on the city, and make it known to the audience that they are recording their videos and living their everyday lives in this city.

##### Living in Rotterdam

A more personal approach to this focus on being in Rotterdam is influencers pointing out that their home is in Rotterdam. This was not only hinted toward, but also mentioned or shown: “Anyway, I am happy to be almost in Rotterdam, or in other words, that means I’m almost home.” Homes can also become symbols for being home in Rotterdam when videos of influencers are frequently recorded inside. Walking in and out of their residences, showing the view from their windows or doors, and discussing the location of one’s home all contributed to this. Such statements and footage attract the attention to the city, and show that the influencer lives in this city, and thus belongs to this group of Rotterdam inhabitants.

##### Being from Rotterdam

Some influencers implicitly or explicitly identified or were identified as being a Rotterdammer in their videos. This can be seen, for instance, when someone addresses a Rotterdam influencer: “Did you know we just came from your hometown? Rotterdam?” In another video, an influencer argues, while wearing a soccer scarf from an opposing soccer team: “By the way, I am from Rotterdam, in case you didn’t know.” In another situation, a Rotterdam celebrity exclaims with a clear Rotterdam accent when talking to about another local influencer: “I think he is a Rotterdammer.” Here, the accent in combination with the exclamation that was an unnecessary remark in the context of the conversation, can be interpreted as not only a strong identification with the city itself by the celebrity, but also the identification of other Rotterdammers as such, and a feeling of connection with other citizens.

#### Culture and Diversity

A second theme that emerged from the data was that of culture and diversity. This theme holds a variety of cultural elements, can be connected to the minority-majority and super-diverse character of Rotterdam, and stresses its urban cultural characteristics that are largely intertwined with this.

##### Citizens and diversity

Citizens and their diversity were commonly presented in the videos. Not only did the influencers themselves reflect a selection of the diverse group of cultures, ethnicities and nationalities that live in Rotterdam, but some also showed ordinary citizens in their videos or interacted with them. This particular focus on ordinary Rotterdammers can be best exemplified by one of the influencers who conducted interviews in the streets of Rotterdam. They addressed their interviewees with a question, often with a silly topic, and had the interviewees’ answer, allowing them to speak and talk about themselves. In another example, an influencer interacts for quite some time with an ordinary Rotterdammer when they give away some of their furniture. By allowing the ordinary Rotterdammers to respond and speak for themselves in the video, they are offered a voice in the videos of these influencers, be it under the control the influencer by means of the questions asked and the editing process. Including ordinary Rotterdammers in videos can be interpreted as a way for the influencers to engage with ordinary Rotterdammers as fellow citizens. The influencers interact with ordinary citizens just like their viewers, and do this in public spaces where their viewers could find themselves in their everyday lives. Even when influencers did not interact with the locals, they provided an image of Rotterdam and its citizens by placing themselves in public spaces recognizable to the Rotterdam viewer from their everyday lives. This shows the viewer that the influencers are present in their environment and that they surround themselves with other ordinary Rotterdammers.

Many of the videos reflected the multitude of diversities, and its complex relationships that characterize the super-diverse city. Both the diversity among the influencers, as well as that among other Rotterdammers in the videos, contributed to this reflection of the super-diverse city. Diversity was mostly present in the videos in the form of nationality, ethnicity, culture, socio-economic background, and, to a lesser extent, a focus on migration. For the sake of this analysis, these elements have been isolated from their complex relationships to each other. Diversity on a national level was often present in forms of mentioning, questions toward, or expressing of one’s national background, such as: “You are a real Surinamese,” “My mother is half Italian,” or, jokingly, “Are you a Turkish Moroccan?” Connecting to this, a diverse set of ethnicities were present in the videos. While this diversity was visually present, ethnicity was seldom discussed. Emphasis on cultural backgrounds was another way in which diversity became apparent, which was mostly expressed by means of cultural elements such as, for instance, playing or performing world music, religious expressions, lifestyle choices, and the various languages spoken. The videos also contained socio-economic diversity. The presence of expensive possessions, ranging from cars and houses, to a relatively luxury lifestyle gave the impression of a high socio-economic status among some of the influencers, which is possibly the result of their work as (successful) influencers. However, not all grew up with such a socio-economic status, which was particularly demonstrated by one of the influencers who receives an all-in holiday as a reward for their hard work on their channel and, overwhelmed with emotions, reveals that they have never been on holiday. Later they say, in disbelief and gratefulness: “I am proud of myself. I’m not allowed to say this, but with all due respect: what [young person from my neighborhood] can say that they have reached [so many] followers? No one.” This demonstrates the socio-economic differences that exist within the city, particularly on a neighborhood level. Finally, migration was implicitly and explicitly mentioned in a few instances. When talking to a girl with a migrant background, one influencer asks: “How long have you been living in the Netherlands?” stressing her migration background. Additionally, when visiting a small town, one of the influencers jokingly asks if they have ever met a beautiful black man, emphasizing the absence of diversity in the rural areas as opposed to the rich diversity in the city. Together, various forms of diversity, which were present in different combinations, represented the complexity of the super-diverse city and its citizens.

##### Language and dialect

Language was a recurring element in many of the videos on various levels. First of all, many different languages were spoken in videos. While the main language of all videos was Dutch, the national language of the Netherlands, words or sentences were spoken in other languages. Sometimes ordinary Rotterdammers in videos were asked to speak their mother tongue: “How would you say I love you and I never want to lose you in Antillean?” Or, in another instance: “How would you say that in Turkish?” Other times, influencers spoke other languages, or emphasized that Dutch was not their first language: “But Creole is my language.” Some of the influencers were also able to understand some of the other languages spoken, indicated by responding in the same language or in Dutch.

Aside from, and perhaps in contrast to, other languages was the Rotterdam dialect and accent. This dialect and accent was mostly spoken by white Dutch Rotterdammers, including some of the influencers and their families. While sometimes it was exaggerated for reasons of humor and mockery, it can be perceived as a clear marker for many that signifies being born and raised in Rotterdam. It was also used to explicitly mark one’s Rotterdam identity. Interestingly, well-known dialect sentences that address the origins from the speaker were playfully used in some instances by non-white influencers to point out their affiliation with the city and culture of Rotterdam. For example, when an influencer accompanied by another Rotterdam celebrity exclaims in dialect: “Are we from Rotterdam? Can’t you hear it?” Despite it not being their dialect in their daily speech, they used this to emphasize their Rotterdam identity.

##### Urban culture

Various typical urban cultural elements were present in the videos. Urban culture may include many (cultural) elements that are important to people’s everyday lives in the city (not to be confused with, or limited to, the music and lifestyle scene also termed *urban*). The most frequent and important examples in the videos related to soccer, as well as hip hop, rap, and other associated cultural elements. These themes are to some extent related to the genre or community of some of the influencers, as demonstrated in the network analysis.

Music was a common element in the videos with hip hop and rap as the most frequent genres. This is in line with our network analysis in which many of the popular local influencers appeared in the *Dutch Urban Music Community*, which was characterized by urban music and cultural diversity. Music, predominantly hip hop and rap, was played and listened to, but was also performed (professionally and non-professionally) and recorded. Moreover, the videos also included (references to) cultural elements that are often associated with hip hop, rap and the associated lifestyle, such as slang, a particular clothing style, or references to life on the streets. This theme was sometimes accompanied and characterized by a dichotomy that touches the theme of diversity on many levels, such as socio-economic position (rich versus poor), ethnic (generally white versus non-white), and of course cultural (dominant versus non-dominant). When two influencers are discussing hip hop, one tells the other: “Did you know I still listen to a lot of Tupac and Biggie? […] I might look like a frat boy, but listen, I’m ‘gangster’.” Here, the influencer seems to hint toward a certain lifestyle that diverges from the way he looks, and expresses a preference for that lifestyle that counters his identity on the basis of his appearance. Such expressions of urban culture might therefore be seen as reflections of the multicultural city, urban inequalities, and, on the other hand, the cultural richness that characterizes the city.

Soccer was a central element to the videos of a number of influencers, as was found in the network analysis, but it was certainly not limited to those within the soccer community. Soccer was a carrier of many other themes, but in doing so also fulfilled its own urban cultural function. Not only was it associated with the local soccer teams, and rivalry with other cities, it can also be seen as a sport available to everyone, despite one’s background. Some of the influencers showed and talked to famous professional soccer players from Rotterdam in their videos. The soccer players often had diverse ethnic, national, or cultural backgrounds, reflecting the super-diverse character of the city. A professional soccer player tells to one of the influencers “At a certain point on a Saturday morning I thought to myself: I just want to play soccer with my buddies,” and continues to tell that he joined his friends at the local soccer team and never stopped playing since. Addressing such locals that became national, if not global, celebrities can be considered a way to reflect how, for some, soccer can play a role in making it in life, or even potentially improve one’s socio-economic status, especially as celebrities are often seen as role models to adolescents. Soccer, which is shown as an accessible sports that can both be played on the field and on the streets, can thus be seen as an urban cultural element that connects many elements of the city, including, but not limited to, urban diversity and the social identity of an urban soccer team.

#### People and Relationships

The social environment of the influencers in relation to their portrayal of the city was the third theme that emerged from the videos. This can be distinguished into two subthemes: celebrities and influencers, and their personal Rotterdam network and family in relation to the city.

##### Celebrities and influencers

In their videos, the influencers were often not the only famous people that could be found. Often times, these influencers had a network of other influencers and celebrities around them that could be considered friends, acquaintances or colleagues. This ranged from working together, to hanging out together in an everyday setting, as well as making videos together. One influencer introduces another: “I am here with an old friend, […] I knew her before YouTube.” Other videos included official collaborations, but also included being on the set of making another media product with other influencers or celebrities, such as television programs, YouTube videos or recording music. Many videos included other Rotterdam celebrities and influencers either by them being present or mentioning them. Interestingly, five out of six influencers appeared in each other’s videos, some more frequently than others. This reflects a Rotterdam network, which is in line with the local network found in the network analysis. Moreover, others of the 18 identified local influencers, as well as influencers that they featured or subscribed to on their channel page, were also present in the videos, again reinforcing the Rotterdam network established in the network analysis. Rotterdam professional soccer players, music artists, and television personalities also frequently appeared in the videos.

Rotterdam influencers in some cases identified or were identified as urban influencers, meaning that they were famous in the city and known by an urban audience. One influencer, when shooting their video far away from Rotterdam, asks people if they know who the influencer is. While the answers are mixed, the explicit question, phrased in a somewhat mocking way, not only seems to stress the urban versus the non-urban, but also seems to indicate that the influencer did not expect those outside of the city to know them. This would in turn indicate that they expect their audience to be primarily urban, as opposed to, for instance, rural. This image was often reinforced by being recognized in a city context. A clear understanding of one’s audience as being urban may then indicate a form of identification with the city as the place where their audience resides, where they are famous, and with which and whom the influencer can identify.

##### Personal Rotterdam network and family in relation to the city

Influencers’ videos also frequently featured, either by means of mentioning or showing, their friends and family living in the same city. Particularly interesting here was the mentioning of friends or family in relation to the city. This ranged from growing up together, to supporting the same soccer team, to them living in the same city. When explaining about their relationships with other influencers and differentiating these work-friends from what they call ‘real’ friends, one influencer explained: “My friends I met mostly here in Rotterdam. I know them since they were kids, and we’ve experienced so much together. That’s where my roots are.” The influencer thus stresses how their local friends with whom they grew up are still very important to their life, stressing their history in Rotterdam. Another influencer shows how their Rotterdam identity is something they want to pass on to their children: “I always wanted to take [my daughter to a Feyenoord game], but she was too young.” Feyenoord is important here, as it is one of the professional soccer teams of Rotterdam. The influencer then goes on to dedicate a whole video to this activity. This focus on one’s personal network of friends and family in Rotterdam symbolizes the entanglement of one’s life with other non-famous Rotterdammers, and sometimes even accentuated their personal history in the city.

#### Urban Symbols

A fourth theme that was found dealt with urban symbols and can be divided into a variety of subthemes: areas, landmarks, teams, events, businesses, and explaining urban symbols.

##### Areas

Areas referred to any mentioning or showing of Rotterdam or its specific neighborhoods, including towns and small cities in the Greater Rotterdam Area. Frequently, the influencers were driving or walking through various parts of Rotterdam. Showing such areas may invite the viewer to recognize the surroundings, and may be perceived as environments that they live their everyday lives in.

##### Landmarks

Landmarks, on the other hand, often included, but were not limited to, the well-known Erasmus bridge, the De Rotterdam building, but also various soccer stadiums, as well as the river, local public transport, and lesser known landmarks. One influencer shows a cruise ship and, behind it, the Erasmus bridge and stresses its size: “A bizarrely big cruise ship. Bigger than the other, higher as well. […] You can barely see the Erasmus Bridge!” Similar to the areas, these landmarks are (the décor of) the sceneries in which the videos take place, offering the possibility for the viewer to recognize this as the environment that they themselves live their everyday lives in.

##### Teams

Teams in most cases referred to soccer teams, including Feyenoord, Sparta, and Excelsior, with the first being dominant. This connects to the network analysis findings where the *Dutch Soccer Community* was among the most important and hosted Dutch soccer teams, including Rotterdam teams. Soccer teams, and especially the fandom surrounding it, can be considered important markers of urban identity – belonging to the city and supporting their team. Influencers visited games, wore logos on clothing, talked and played with (professional) players, and so on. This theme connected to other urban symbols such as events, landmarks, logos, but also celebrities and influencers, as well as rivalry with other (urban) identities.

##### Events

The events in Rotterdam that were shown or mentioned ranged from sports events, such as soccer games and the Rotterdam Marathon, to other local events, such as the International Film Festival Rotterdam and Trek Festival. One influencer explains as they drive away from their home and, on the way, show footage of the marathon event: “Today, the marathon of Rotterdam takes place, or, in other words, it’s very crowded in the city.” These events are happening in the lives of influencers and their Rotterdam audience at the same time. Moreover, in many instances, these events can be linked back to the previous three urban symbols of areas, landmarks and teams.

##### Businesses

Some influencers also mentioned or visited local Rotterdam businesses of different sizes – ranging from restaurants, to hairdressers, to the Feyenoord shop. For example, influencers went to eat at a local restaurant, named the restaurant, and showed the food. Sometimes the employees or owners were shown as well. Moreover, they helped their fellow Rotterdammers by promoting these businesses. Such collaborations can be considered crucial and common in terms of expending each other’s reach, as well as influencer marketing related activities. Not only are such businesses owned by fellow Rotterdammers, these businesses may also be recognizable to the viewers and might be places they visit or pass by now and then.

##### Explaining urban symbols

Urban symbols were in some occasions also explicitly named or explained by influencers. By pointing out the urban symbols or giving an explanation about the urban symbol, sometimes in relation to living in Rotterdam, they seemed to aim to educate the viewer on the city of Rotterdam, and position themselves as locals or at least very familiar with the city. An example of this is when an influencer shoots a video while traveling from one side of the city to the other, and on the way they describe all the famous landmarks and their location in the city: “I am acting as a tour guide,” they proclaim. Later they emphasize their local knowledge and, seemingly, their everyday living experience: “I just arrived at Leuvehaven [station], that stop is of course famous for Rotterdammers and [the announcement voices] always say: Leuvehaven station, Eyehospital.” Such explanations of urban symbols thus function as an extra emphasis, and can be perceived to connect the environment to the (knowledge of) the influencer.

#### Comparisons With and Reflections on Other Identities

As a final theme we found that the images of Rotterdam, its citizens, and urban identity were also established next to images of their opposites. The most important contrasts found were Amsterdam, other cities, and the comparison of urban versus rural.

##### Amsterdam

The rivalry and comparison between Amsterdam and Rotterdam is nothing new in the Netherlands, and it was therefore not surprising that this was present in the videos. As the two biggest cities in the Netherlands, they have a long history of competition and rivalry on many fields. It was therefore surprising that Amsterdam was frequently returning in a neutral or positive light in the videos of the Rotterdam influencers. One of the influencers even expressed wanting to move to Amsterdam: “I was just driving, and I thought to myself: I like Amsterdam so much. So I think I will be moving here before summer.” Many of the influencers frequently visited Amsterdam for (influencer) work-related reasons. Despite the well-known rivalry between the cities, Amsterdam therefore seemed key to the lives of the majority of these influencers. The rivalry between these cities was, however, not entirely absent from the videos, but in a few instances presented itself in innocent, sometimes slightly teasing ways. It was mostly found in the context of soccer, and the rivaling teams Feyenoord (Rotterdam) and Ajax (Amsterdam). One influencer sees Ajax bedsheets and playfully and mockingly proclaims: “What do I see over there? I see a gnome! Uh, I mean the Ajax logo.” Another example is when a Rotterdam influencer explains that he needs to go to Amsterdam, and a Rotterdam celebrity quasi-offendedly yells: “What?! Amsterdam!?” These instances show the presence of rivalry, but were all embedded in a context that limited the negative implications of such statements. The innocent rivalry, and its particular presence in soccer, was even explained by one influencer who argued: “I fanatically support Feyenoord, but that does not mean I hate people from Amsterdam or people who support Ajax.” Such a statement can be interpreted as rather remarkable, since the rivalry between these two soccer teams has been characterized by hatred and intolerance among some of their supporters. While a rivalry with Amsterdam is still present and Rotterdam influencers seemed to slightly differentiate themselves from this city, it was not a strong contradiction that defined Rotterdammers as non-Amsterdammers, which makes this a surprising finding. However, this innocent approach to the rivalry may have various explanations. On the one hand, it seemed as if the job of an influencer to a certain extent entailed being part in the Amsterdam media environment and network, with many of the studios, companies, agencies and MCNs located in the area. On the other hand, it might also be a matter of commercial self-interest, where choosing sides or excluding or alienating part of one’s audience can only do harm to the number of views and followers.

##### Other cities

Other cities were also presented in the videos, but in these cases less rivalry was present. This was not limited to cities in the Netherlands. These cities ranged from very large international cities, to smaller Dutch cities. Mostly the cities were only named, however, a few were also shown, including and ranging from Amsterdam and The Hague, to Katwijk and Liverpool. Particularly the mentioning and showing of non-Rotterdam soccer teams offered a place for other (urban) identities to be present. One of the influencers frequently shows other soccer teams, players and training fields in their videos, and sometimes even plays against players from a variety of other predominantly Dutch soccer teams. However, despite the game element, very little rivalry is present on an identity level. Sometimes a distant admiration was even present, as when approaching the stadium of Liverpool, one influencer exclaimed: “We are here! Look at that: the beating heart of Liverpool!” At the same time, Rotterdam was also, in select cases, distinguished from other cities. One influencer proclaims as they are showing a number of Rotterdam landmarks: “Sometimes I forget how beautiful Rotterdam is and how much it differs from other cities.”

##### Urban versus rural

A final, relatively infrequent but quite powerful dichotomy found in the videos was the comparison between the urban versus the rural. People from the rural provinces of the Netherlands were generally seen as inherently different. Differences between the urban and the rural were often made clear by means of explicit mentioning of these differences, such as a lack of a multicultural environment, urban culture and the conveniences of city life. One of the influencers asked someone from a rural area if they had a public transport card, and, after a negative answer, the influencer emphasized the absence of public transport in rural areas. Moreover, these differentiations included a lot of stereotypical remarks about the countryside, such as the idea that all rural citizens were farmers, the mocking of the rural dialects, and rural towns as places where no one wants to live. One of the influencers mockingly proclaims: “We in Rotterdam West, South, North, East – we have it all. But in this [small town], they have a bakery, too!” Another combines many rural stereotypes to describe being in a rural area: “We would be sitting in between cows and pregnant donkeys, and fun and all, and we could inseminate and everything that comes with it.” All in all, this clearly articulated distinction between the urban and the rural put an emphasis on what it means to live inside a city – particularly that of Rotterdam.

## Discussion

This research investigated how the local influencers most popular among local adolescents of Rotterdam are affiliated with each other, how their channels relate to the wider media ecology, and continued by identifying to what extent and how the content published by these local influencers relates to the super-diverse city and its citizens.

The results of the network analysis and the thematic analysis showed the connection between the YouTube ecology and the video content in the presentation of the city. The affiliations between local influencers found in the network analysis were also reflected in the thematic analysis, with five out of six influencers appearing in each other’s videos, next to other (local) influencers from the network. This twofold local affiliation emphasizes the importance of the local network. Furthermore, the communities found on the basis of YouTube’s recommender system showed that many of the influencers were recommended according to themes that reflected cultural aspects of the city that also emerged from the thematic analysis: urban music lifestyle and soccer. Combined, the findings demonstrate a number of overall findings.

### The City as a Recognizable Framework for Strengthening Urban Identity

The network analysis and thematic analysis showed that the city can be understood as a recognizable framework for negotiating and strengthening adolescents’ urban identity. The city and its citizens were mostly present in the background of the videos or mentioned in passing. Many of the videos took place in the city (see *Being and Living in the City*), showing the local scenery (see *Urban Symbols*), engaging with local influencers, celebrities or ordinary Rotterdammers with diverse backgrounds (see *People and Relationships* and *Culture and Diversity*), and presenting (sub-)cultural elements that can be considered strongly connected to the city (see *Culture and Diversity)*, and, additionally, a local network of affiliations could be found in terms of the YouTube ecology in which these influencers find themselves.

Through the position of the influencers within the context of Rotterdam, Rotterdam adolescents may find themselves recognizing many aspects related to the city and its citizens, touching on the spatial experience of the city (Proshansky et al., 1983; Lalli, 1992) as well as symbolic elements that are affiliated with the city (Proshansky et al., 1983; Lalli, 1992; Spaaij, 2008; Belanche et al., 2017). In line with findings by Schwartz and Halegoua (2015); Belanche et al. (2017), and Saker (2017), the city was often represented through symbols that represent the Rotterdam environment and by marking locations that are symbolic to the city, both of which may be easy to recognize for local adolescents. Next to spatially symbolic elements, cultural elements may also play an important role. For instance, Rotterdam soccer teams, as found both in the videos as well as the related channel communities, may provide a collective identity through shared fandom (Murrell and Dietz, 1992; Spaaij, 2008), as soccer teams are often connected to a city, functioning as a vehicle for a local identity, and rivalry between particular teams helps to strengthen collective identity through the feeling of us versus them (Spaaij, 2007, 2008; Shobe, 2008). Moreover, the presence of the Rotterdam accent and dialect adds an extra possibility for recognition, as dialect can be seen as a form of expressing one’s place identity (Johnstone, 2011), and the Rotterdam accent is generally recognized in the Netherlands. Together, these representations of the city provide the décor for the videos and meaningful symbolical elements, which may increase the perceived affiliation between the influencer and the city. Next to that, it can be understood as a mostly positive representation and evaluation of the city (Lalli, 1992; Belanche et al., 2017), may help strengthen a collective identity (Murrell and Dietz, 1992; Spaaij, 2008), and, in turn, may increase the likelihood of young Rotterdammers being able to identify with these influencers.

This affiliation with the city was further reinforced when influencers mentioned their locations, sometimes almost as if checking in, which previous research has argued can be understood as a form of identity construction based on place (Schwartz and Halegoua, 2015; Saker, 2017). Many of the influencers showed their Rotterdam homes and some even straightforwardly identified themselves as Rotterdammers, signifying that they identify with the city they live in and feel a sense of belonging to the city and its citizens, which can be perceived as a direct link to urban identity (Lalli, 1992; Belanche et al., 2017). Moreover, by including ordinary Rotterdammers in their videos, the influencers reinforced the creation of authenticity, approachableness, and parasociality, particularly for a Rotterdam audience, which is key to the success of the influencer and may therefore strengthen their function as role models (Abidin, 2017; Burgess and Green, 2018). Together, this can be perceived as a way in which they establish their affiliation with the city, which may help adolescents recognize the influencers as ‘Rotterdammers’ and likeable role models, and provides them with recognizable elements that signify a sense of belonging to the city that they might use in their own negotiation of their urban identity.

A final way in which the influencers supported an urban identity strengthening framework was through the negotiation of what Rotterdam was not – a comparing and contrasting between Rotterdam and other cities or areas. Influencers included elements that clearly differentiate Rotterdam from other cities or areas, by means of the cities themselves, teams, or other themes such as contrasting the urban against the rural, aiding the negotiation of Rotterdam identities (see *Urban Symbols* and *Other Identities*) (Proshansky et al., 1983; Lalli, 1992; Belanche et al., 2017). Interestingly, rivalry between cities seemed generally absent or was only playfully indicated, which was surprising due to the well-known rivalry between Amsterdam and Rotterdam, in particular their soccer teams (Spaaij, 2008). No strong position was taken, which can be possibly explained by the large audience that these influencers address that exceeds the boundaries of the city. This connects to our finding that their overarching related channel communities were clearly Dutch (versus international communities), but not limited to Rotterdam communities. The videos may thus contain symbols, contexts and situations highly recognizable to a Rotterdam audience, possibly reinforcing an existing urban identity, but non-alienating to those outside of this context, which in turn may support the influencers’ authenticity and accessibility aiding the parasocial relationship with all of their viewers.

### A Reflection of Rotterdam as a Super-Diverse City

Rotterdam’s super-diverse character also plays an important role in the presentation of the city. First of all, the influencers themselves seemed to have different ethnicities, cultural, and socio-economic backgrounds, representing their Rotterdam audience quite well. This goes against the idea that media production is limited to those with a privileged background (Brake, 2014; Usher, 2018). However, it could be argued that the glamorous lifestyle differed from that of many ordinary citizens, as is the case with many celebrities. This glamorization of urban life might present a too optimistic view of everyday life in the city.

The representation of the city as super-diverse was also reflected in what and who these influencers showed (see *Culture and Diversity*). From the overwhelming diversity of ethnicities, nationalities, cultural backgrounds, as well as languages and (sub-)cultures, we can see that the presentation of Rotterdam exceeds a white-dominated Dutch culture, and is a clear indication of the Rotterdam super-diversity, especially due to their complicated relationship with other elements such as socio-economic status, and the various waves of migration from various countries throughout the history of Rotterdam (Vertovec, 2007; Jennissen et al., 2018; Statistics Netherlands, 2018; Scholten et al., 2019). While the extent to which this (super-)diversity was present differed between influencers and their videos, the presence of a great diversity on various aspects may at the very least be said to start to reflect the intertwined complexity of diversities that characterizes the super-diverse city (Vertovec, 2007). The diverse backgrounds of the influencers and celebrities, reinforced by the diversity among ordinary Rotterdammers that appear in their videos, may provide a diverse pool of potential role models with whom these children with different backgrounds may identify themselves through their similarities (Gleason et al., 2017; Valkenburg and Piotrowski, 2017).

Moreover, super-diversity was also reflected in the centrality of Dutch hip hop and soccer in the related channel communities and the videos of the influencers (see *Culture and Diversity*). These themes speak to the young target audience and hold important cultural symbolism that is key to understanding urban identity in Rotterdam (Bennett, 1999; Alim and Pennycook, 2007; Spaaij, 2008; De Groot, 2019). Hip hop and rap music are known to often deal with (local) urban issues that are central to multicultural and super-diverse cities (Bennett, 1999; Alim and Pennycook, 2007; De Groot, 2019). The urban relevance of soccer could be found both in its approachable element, being available to everyone despite socio-economic status, and at the same time in its symbolism in terms of the local teams. Together these many elements tie together and begin to reflect the various levels and complexities of diversity that exist within the city of Rotterdam.

### The Importance of the Local Influencer Network

Both the network analysis and the thematic analysis showed the importance of the social environment in identification with and creating a sense of belonging to the city. The videos as well as the network reflected the role of personal and professional networks that are central to the success of influencers. While the content of the influencers’ videos varied greatly, as did the related channel communities in which they were found, the influencers, particularly those who were connected by one or two steps in our network analysis, could regularly be found in each other’s videos (see *People and Relationships*). While some influencers were clearly collaborating frequently in terms of video making, others appeared in more informal settings. This was true for those within the same community, but also those outside of it. This network and mutual support among influencers from the same city can be understood not only in terms of informal connections, but may also be the result of the MCNs that these influencers are affiliated with (Lobato, 2016; Johnston, 2017). Nevertheless, the affiliation between local influencers strengthens a sense of a network of Rotterdam influencers that engage with each other. Adolescents find themselves living in the same city as a group of their media idols, which may potentially strengthen their collective identity, as well as provide them with a pool of role models from which adolescents may borrow in their (urban) identity construction. This centrality of urban identity is further emphasized by the presence of the influencers’ friends and families and their personal history in the city, which can be understood in terms of the importance of the idea of a place related social community and one’s perceived history in the city (Proshansky et al., 1983; Lalli, 1992; Hidalgo and Hernández, 2001; Kim and Kaplan, 2004; Belanche et al., 2017; Van Eldik et al., 2019).

### Limitations and Future Directions

This project has combined methods of quantitative network analysis with qualitative thematic analysis, based on earlier results from a survey study. The results of these analyses supported each other, and the multi-method approach of this research helped to understand and combine the findings on the content of the videos in light of the current online media ecology. By basing the choice of influencers on the previous survey results, the findings of this research reflect content of influencers that were truly popular among and were recognized as Rotterdammers by the young participants, instead of picking influencers on the mere basis of views or subscriber counts. The results of this research help further investigating the role of influencers in identity construction, particularly in light of adolescents’ lives in the super-diverse city, and demonstrate the different forms and messages that the content can hold. In light of earlier findings that showed that social media engagement and local influencers play a role in the construction of urban identity, a sense of belonging and even, through this, self-esteem (Van Eldik et al., 2019), the current findings fill the gap with respect to what the content of such local influencers’ videos actually entail.

This study also has a number of limitations. Firstly, in May 2019, YouTube deleted its related channel network from the YouTube interface, as it was deemed not frequently enough used (YouTube, 2019). These suggestions are therefore no longer visible on the influencers’ channel pages. Secondly, the qualitative thematic analysis was based on a selection of six influencers, and while effort was made to include different genders and cultural backgrounds whilst sticking to the popularity as indicated by the empirical findings of the survey, it was outside the scope of the research to make distinctions on the basis of these demographic characteristics. Future research might benefit from taking gender, cultural background, but also online community or genre into account for an even more nuanced image, or specific results for a specific group of viewers. Finally, as role models influencers may also present unrealistic identities that could be harmful to adolescent identity development alongside and intertwined with urban identity, such as gender, racial, and socio-economic stereotypes. Future experimental studies are needed to investigate the effects of these local influencers as role models on adolescents, focusing on what is obtained from watching the videos.

All in all, this research has contributed to the literature on urban identity presented in the online environment, both in terms of the network as well as the content of influencers preferred by adolescents. Our results therefore indicate that influencers, in their role as role models, may play an important part in adolescents’ urban identity construction and, in turn, play a role in empowering them through the representation of the super-diverse city in which they find themselves and of which they are part.

## Data Availability Statement

The datasets for this article are not publicly available due to the copyright and for reasons of protecting the anonymity of the data. Requests to access the datasets should be directed to AE, vaneldik@eshcc.eur.nl.

## Author Contributions

AE, JK, and JJ designed the study. AE and RL conducted the network analysis. AE conducted the thematic analysis together with an external coder. AE, RL, JK, and JJ wrote and approved the final version of the manuscript.

## Conflict of Interest

RL was employed by Center for Media & Health. The remaining authors declare that the research was conducted in the absence of any commercial or financial relationships that could be construed as a potential conflict of interest.
