# Supplementary material for: Urban Influencers: An Analysis of Urban Identity in YouTube Content of Local Social Media Influencers in a Super-Diverse City
Source: Front Psychol. 2019 Dec 19;10:2876. doi: 10.3389/fpsyg.2019.02876 (PMC6930894; doi:10.3389/fpsyg.2019.02876)
Supplement: Supplementary file 1 [file Table_1.pdf]

Table 1. An oversight of the final codes. The colors represent codes and their sub-codes.

Identifying by means of location and city	*Being in Rotterdam* (Pointing out location Rotterdam)		Whenever they point out that they are currently in, going to, or were just in Rotterdam.
	Living in Rotterdam (Stress on home being in Rotterdam)		Whenever they point out that they are at home, that their home is in Rotterdam, or when it becomes clear that they are home in Rotterdam, or that they live in Rotterdam.
	Being from Rotterdam	Rotterdam Identity (implicit)	Implicitly expressing their or others' urban identity.
		Rotterdam Identity (explicit)	Explicitly expressing their or others' urban identity.
Culture and Diversity	Language (diversity)	Diversity	Languages other than Dutch being spoken or being requested.
		Rotterdam Dialect	People speaking with or attempting to copy a Rotterdam dialect or accent. May include Rotterdam stereotypical behaviors, such as being very direct, and having a very cruel or rude sense of humor.
		Slang	Any slang or language associated with the urban landscape.
	Urban Culture	Hiphop / rap / other	Any mentioning or showing of hip hop, rap, or other culture often associated with an urban landscape. Includes recording, performing, listening to, and so on. Might also include other elements such as clothing or famous symbols.

		Soccer	Any mentioning or showing of soccer, teams, related landmarks, related influencers, etc.
	Citizens	Focus on regular Rotterdammers	When non-famous people in Rotterdam are deliberately focused on, such as in the case of street interviews.
		Diversity: National	Any mentioning, question towards, or expression of one's nationality / national background.
		Diversity: Cultural	Any cultural expressions, such as music specific to a culture, one's language, religion, or other cultural elements. This can be shown, mentioned, or asked about. Includes explicit stress on Dutch culture.
		Diversity: Ethnic	When ethnicity (or diversity between ethnicities) is mentioned, discussed or very clearly shown or emphasized. Possible by means of contrast.
		Diversity: Socio-economic	When differences in socio-economic status become clear – spoken or shown. This can be in terms of poor versus rich, but may also include job (opportunities) or other expressions of socio-economic status.
		Diversity: Migration	Any discussion of or hint towards migration. This may include the issues of migration, but can also focus on individuals making it, sometimes implicitly, clear that they migrated. It may also possibly include the discussion of the lack of migration in an area.
Social / Urban Network	Celebrities and Influencers	Rotterdam	All Rotterdam celebrities and influencers shown or mentioned. Includes when they identify themselves or are identified by others as influencers.
		Rotterdam: identifying as an urban influencer	Whenever a Rotterdam influencer shows or makes a statement that implies in a way that they consider themselves an urban influencer (known by people in the city).
		Other influencer / celebrity	All non-Rotterdam celebrities and influencers shown or mentioned.

		Focus on influencer network	Whenever it becomes clear that the influencer has a network of other celebrities or influencers surrounding them. This can be in mundane ways, such as hanging out in a casual manner.
	Personal Rotterdam Network		Any focus on their personal Rotterdam friends or acquaintances other than influencers.
	Family and the City		Any mentioning of family in relation to the city.
Urban Symbols	Area		Any mentioning or showing of Rotterdam or its specific neighborhoods etc. This includes surrounding cities that are generally considered to be part of the Rotterdam area.
	Landmark		Any mentioning or showing of Rotterdam landmarks. (Includes public transport).
	Team		Mentioning or showing any Rotterdam team. (Sparta, Excelsior, Feyenoord)
	Event		Any event associated with Rotterdam, such as sports events, festivals, or other events.
	Business		Any mentioning or showing of businesses in Rotterdam.
	Explaining Urban Symbols		Whenever Rotterdam urban symbols are explained or further identified. Often this means it is further related to the (history of/other landmarks of/events in) the city.
Other Identities	Amsterdam		Includes any mentioning of being, going to, or having been in Amsterdam. Also includes discussions about Amsterdam. Includes its teams, landmarks, and other urban symbols.
		Positive	Positive remarks about Amsterdam, sometimes in contrast to Rotterdam.
		Negative	Negative remarks about Amsterdam, sometimes in contrast to Rotterdam.

		Rivalry	The rivalry between Rotterdam and Amsterdam, which can be found in terms of the city itself, but also in soccer. Also includes comparisons being made between cities, clearly distinguishing or differentiating it from Rotterdam or other cities.
	Other cities		Mentioning or explicitly showing other cities, including their urban symbols such as teams, or important landmarks.
		Rivalry/comparison	Any rivalry between other cities and Rotterdam. Also includes comparisons being made between cities, clearly distinguishing or differentiating it from Rotterdam or other cities.
	Urban versus Rural		Making a(n indirect) comparison between the city and the provinces. (Includes, for instance, Drenthe, Groningen, etc.)
		Rural Stereotypes	When typical rural stereotypes are being expressed implicitly or explicitly.
	Opinion about Rotterdam	Positive	Any expression of a positive opinion about Rotterdam.
		Negative	Any expression of a negative opinion about Rotterdam.
